# Should public health interventions aimed at reducing childhood overweight and obesity be gender-focused?

**DOI:** 10.1186/1471-2458-10-340

**Published:** 2010-06-14

**Authors:** Aline Simen-Kapeu, Paul J Veugelers

**Affiliations:** 1 School of Public Health, University of Alberta, Edmonton, Canada

## Abstract

**Background:**

Overweight in childhood is a major public health concern that calls for immediate preventative action. An increasing number of reports suggest that gender specific approaches to prevention may be more effective. However, there is a paucity of information to guide gender-sensitive health promotion and population health interventions for the prevention of overweight in childhood. In the present study, we sought to determine gender-differentials in overweight and underlying behaviors, nutrition and physical activity, among pre-adolescents in Alberta, Canada, to inform the discussion on gender-focused interventions for chronic disease prevention.

**Methods:**

In 2008, we surveyed 3421 grade five students and their parents of 148 randomly selected schools. Students completed the Harvard food frequency questionnaire, questions on physical activities, and had their height and weight measured. Parents completed questions on socio-economic background and child's lifestyle. We applied multilevel regression methods to assess gender differentials in overweight, nutrition and physical activity.

**Results:**

Overall, the prevalence of overweight was slightly higher among boys (29.1%) than girls (27.9%) with more pronounced differences in towns and urban geographies. Boys reported to be much more physically active relative to girls (OR = 2.12, 95% CI: 1.73-2.60). Diets of boys, relative to those of girls, reportedly constituted more fat and were less likely to meet the recommendation of 6 daily servings of vegetables and fruits (OR = 0.81, 95% CI: 0.71-0.93).

**Conclusion:**

Our findings confirm the existence of gender differences in physical activity and nutrition, and support gender-focused health promotion whereby priority is given to physical activity among girls and to healthy eating among boys.

## Background

The prevention of childhood overweight and obesity is a public health priority in developed countries [1]. Unhealthy diets and physical inactivity are the established determinants of the rising prevalence of overweight and obesity in childhood [2,3]. Research of these determinants has become increasingly important due to the increasing recognition of the need for preventative actions. For example, childhood overweight has been reported to vary between geographies, with higher prevalence rates observed in rural areas relative to urban areas in most developed countries, suggesting that priority for programs should be given to children and youth residing in rural areas [4].

The etiology of overweight and obesity among girls and boys may be different due to biology (sex differences) as well as society and culture (gender differences) [5,6]. Various studies have examined the importance of gender for overweight and obesity in childhood [5,7-9], some reported no gender-difference [10,11], some reported higher overweight prevalence among boys [12,13] whereas other reported higher overweight rates among girls [14,15]. Some recent studies showed gender differentials with respect to the behavioral determinants of overweight including calorie intake [16-19] and physical and sedentary activities [20-23]. Girls were shown to pay more attention to foods as a way to influence health and to meet nutritional recommendations, and boys reportedly ate more fast foods [16,17]. Adolescent girls reported less physical activity, fewer active role models, more barriers and less perceived benefits to physical activity relative to boys [20,22].

Consideration of gender differentials in public health interventions may have positive effects on their effectiveness [24]. Gender mainstreaming, an approach where no considerations is given to gender specific needs in the development and implementation of policies, programmes and projects, may therefore be less effective and miss the aim of gender equity. In fact, it has been suggested that gender mainstreaming in health promotion may contribute to gender-differences in health behaviors [5]. Even though knowledge of gender-differences in childhood is increasingly available, more research is important to providing guidance to program planners and public health decision makers with a mandate in chronic disease prevention. With the scarcity of gender studies in the field of childhood overweight, we sought to assess gender-differentials in overweight, physical activity, nutrition and health behaviors among pre-adolescent children in the province of Alberta, Canada, to bring more insights into the discussion on gender-focused interventions for chronic disease prevention.

## Methods

### The Survey

The Raising Healthy Eating and Active Living Kids in Alberta survey http://www.REALKidsAlberta.ca aims to evaluate a comprehensive initiative by Alberta Health and Wellness to promote healthy body weights among children and youth through surveys in 2008 and 2010 among grade five students who are primarily 10 or 11 years of age. The present study reports on the 2008 observations. The study employed a one-stage stratified random sampling design. The sampling frame include all 1505 schools with grade five students, with the exception of private schools (4.7% of all Alberta children attend such schools), francophone schools (0.6%), on-reserve federal schools (2.0%), charter schools (1.7%), and colony schools (0.8%) [25]. Schools were stratified into three geographies: 1) urban: Calgary and Edmonton; 2) towns: other municipalities with more than 40,000 residents; and 3) rural: municipalities with less than 40,000 residents. Schools were randomly selected within each of these strata to achieve a balanced number of students in each stratum. Of the 184 invited schools, 148 (80.4%) participated in the study. These schools were attended by 5594 grade five students who received an envelope with a parent survey and consent form to take home. Of the 3758 (67.2%) students that returned completed consent form to school, 3645 (97.0%) had received parental consent to participate. Of these students, 3407 were present when evaluation assistants visited the school to conduct the survey, 6 students declined to participate, and 20 absent students completed and mailed their surveys, resulting in 3421 participating students (61.2% of total student population in those schools). These students completed the Harvard Food Frequency Questionnaire for Youth and Adolescents (FFQ) which is suitable for grade five students [26]. Students further completed a short survey on activities and behaviors, and had their heights and weights measured.

### Measurements

Standing height was measured by trained evaluation assistants to the nearest 0.1 centimeter without shoes using stadiometers and body weight to the nearest 0.1 kilogram on calibrated digital scales. Body mass index was calculated by dividing weight (in kilograms) by height (in meters) squared. Overweight (including obesity) and obesity were defined using the body mass index cut-off points for children and youth by the International Obesity Task Force [27].

Parents and students responded to activity questions on: a) travel to and from school; b) time spent to get to and from school; c) frequency of child's activities outside of school hours; d) activities at morning and lunch recess in the past seven days; and e) frequency of involvement in sports and physical activities in the past seven days. These questions, totaling 29 items, were by large adopted from the Physical Activity Questionnaire for Children (PAQ-C) which has previously been validated and demonstrated high reliability [28]. The 29 items were the basis of a composite score ranging from 1 to 6. Students with a score exceeding 3 were classified as 'physically active'. Students also provided answers on the frequency (< 3 times/week or ≥ 3 times/week) during which they were engaged in physical activities with a coach ("sports with coach"), without a coach ("sports without coach"), or with parents ("sports with parents") out of school hours. Parents answered questions on the number of hours (≤ 2 hours/day or > 2 hours/day) that their child spent playing videos games, watching television and using the computer out of school hours ("screen time"). These questions have been previously validated and were adopted from the National Longitudinal Survey of Children and Youth [29].

Based on students' responses to the FFQ and nutrient information from Canadian food tables [30], we calculated the nutrient intake for each of the participating students. Specifically, we calculated percentage of energy in their diets originating from dietary fat, and calculated whether students complied with Canadian guidelines of 6 servings of vegetables and fruits per day [31]. The questionnaires for students further included 6 questions on types of foods they would eat or purchase (see table 1).

**Table 1:** Characteristics of grade five students in Alberta, Canada

	Prevalence	Boys	Girls	*p *value
**Body weight status**				
Overweight	28.54	29.13	27.98	*0.50*
Obese	6.9	7.57	6.27	*0.16*
**Parental educational attainment**				
Secondary graduation or less	26.54	25.72	27.30	
Postsecondary or college diploma	39.91	38.44	41.29	
University degree	33.55	35.84	31.41	*0.04*
**Household income**				
< $ 50,000	23.37	23.79	22.97	
$ 50,001-$ 75,000	17.48	16.46	18.45	
$ 75,001-$ 100,000	22.19	22.51	21.89	
> $ 100,000	36.96	37.24	36.69	*0.73*
**Geographic location**				
Urban	47.33	47.85	46.84	
Small town	16.21	15.27	17.11	
Rural	36.45	36.88	36.05	*0.33*
**Physical activity**				
Being physically active (PAQ C score > 3)	26.04	31.97	20.43	<0.01
Engaged in sport activities without coach ≥ 3 times/week	38.56	44.47	33.0	<0.01
Engaged in sport activities with coach ≥ 3 times/week	15.72	18.14	13.46	<0.01
Engaged in sport activities with parents ≥ 3 times/week	8.09	7.47	8.68	0.24
Screen time ≥ 2 hours/day	16.27	16.34	16.20	0.90
**Nutrition**				
Buy soup, sandwiches or burritos at school ≥ 3 times/week	12.68	13.62	11.79	0.05
Buy snacks like donuts, candy, chocolate bars and chips at school ≥3 times/week	8.78	9.22	8.37	0.42
Eat food from fast food restaurant ≥ 3 times/week	39.73	42.31	37.31	< 0.01
Eat convenient food ≥ 3 times/week	43.48	45.71	41.37	0.02
Eat fried food at home ≥ 3 times/week	54.4	56.30	52.61	0.05
Eat fried food away from home ≥ 3 times/week	41.4	41.63	40.69	0.61
Mean % energy from fat†	27.56	27.81	27.32	< 0.01
Consumption of ≥ 6 servings of vegetables and fruits/day	26.68	24.72	28.53	0.02

*Significant gender differences (P < 0.05): χ^2 ^-tests were used for all outcomes except for '% energy from fat' where a t-tests was used. All estimated were weighted to represent population prevalence.

† Percentage of dietary energy originating from dietary fat

Finally, the questionnaire completed by parents provided information on household income (≤ $50,000; $50,001-$75,000; $75,001-$100,000 and > $100,000) and parental education (secondary school or less, community college and graduate university).

### Statistical analysis

We applied multilevel logistic regression methods to assess gender differences in overweight, physical activity and nutrition while acknowledging the nested structure of the data whereby student observations nest within those of schools. All analyses were weighted to account for the design effect such that estimates apply to the grade five student population of Alberta. We adjusted for the confounding potential of parental education attainment, household income and residency. Analyses pertaining to dietary outcomes ('having 30% or more of dietary energy from dietary fat' and 'eating 6 or more servings of vegetables and fruits daily') were further adjusted for calorie intake as is recommended for FFQ data [32]. Missing values for parental education attainment, household income were treated as separate covariate categories. Stata Version 10 (Stata Corp, TX, USA) was used to perform the statistical analyses. This study, including data collection and parental informed consent forms, was approved by the Health Research Ethics Board of the University of Alberta.

## Results

Our study included comparable participation of boys (48.6%) and girls (51.4%). The prevalence of overweight, including obesity, was higher among boys (29.1%) than girls (27.9%) although the difference was not statistically significant (Table 1). Among grade five students in Alberta, 26.0% was reportedly physically active. This was statistically significant differed for boys (32.0%) and girls (20.4%). Relative to girls, boys reported to engage more in sports with a coach (18.1%) and in sports without a coach (44.5%). No substantial differences were observed in the reporting of screen time (Table 1).

Boys consumed an estimated 1955 kilocalories per day and girls 1859 kilocalories per day. Table 1 shows that, relative to girls, boys had reportedly more energy from dietary fat and were less likely to meet the recommendation of 6 or more servings of vegetables and fruits per day.

Table 2 depicts the probability of boys, relative to girls, to be overweight, to be physically active and to eat healthy. When adjusting for parental educational attainment and household income, boys residing in towns were estimated to be 40% more likely to be overweight than girls (table 2: OR = 1.40). Relative to girls, boys reported to be more physically active (OR = 2.12). With respect to physical activity, relative similar estimates were observed in urban areas (OR = 2.10, 95% CI: 1.50-2.94), towns (OR = 2.48, 95% CI: 1.62-3.81) and rural areas (OR = 2.00, 95% CI: 1.49-2.68).

**Table 2:** Probability of being overweight or engaging in physical activity and nutrition behaviors of boys relative to girls

	unadjusted		adjusted	
	OR	95% CI	OR	95% CI
**Overweight**				
In urban areas	1.15	0.85 - 1.58	1.18	0.85 - 1.64
In towns	1.38	1.03 - 1.34	1.40	1.05 - 1.86
In rural areas	0.81	0.64 - 1.02	0.82	0.65 - 1.03
				
**Physical activity**				
Being physically active (PAQ C score > 3)	2.09	1.71 - 2.55	2.12	1.73 - 2.60
Engaged in sport activities without coach ≥ 3 times/week	1.65	1.40 - 1.95	1.68	1.42 - 1.98
Engaged in sport activities with coach ≥ 3 times/week	1.47	1.15 - 1.86	1.45	1.14 - 1.84
Engaged in sport activities with parents ≥ 3 times/week	0.84	0.67 - 1.06	0.86	0.65 - 1.09
Screen time ≥ 2 hours/day	1.01	0.81 - 1.25	1.02	0.83 - 1.26
				
**Nutrition**				
Buy soup, sandwiches or burritos at school ≥ 3 times/week	1.28	1.00 - 1.66	1.27	1.00 - 1.63
Buy snacks like donuts, candy, chocolate bars and chips at school ≥3 times/week	1.11	0.83 - 1.47	1.10	0.82 - 1.46
Eat food from fast food restaurant ≥ 3 times/week	1.22	1.05 - 1.42	1.22	1.05 - 1.43
Eat convenient food ≥ 3 times/week	1.19	1.00 - 1.41	1.20	1.01 - 1.42
Eat fried food at home ≥ 3 times/week	1.17	0.98 - 1.40	1.18	1.00 - 1.41
Eat fried food away from home ≥ 3 times/week	1.03	0.86 - 1.24	1.04	0.86 - 1.25
% energy from fat exceeds 30%†	1.63	1.16 - 2.29	1.68	1.19 - 2.35
Consumption of ≥ 6 servings of vegetables and fruits/day	0.81	0.71 - 0.93	0.82	0.71 - 0.96

* Odds ratio (OR) 95% confidence interval (CI). Adjusted risk estimates were adjusted for household income, parental education and residency. '% energy from fat exceeds 30%' and 'Consumption of ≥ 6 servings of vegetables and fruits/day' were further adjusted for calorie intake.

† % energy from fat exceeds 30%: represents the probability that more that 30% of dietary energy originating from dietary fat

Relative to girls, boys were also more likely to report to eat food from a fast food restaurant (Table 2). Boys' diets, relative to those of girls, were more likely to have 30% or more of its energy to be from dietary fat. This gender differential was most pronounced in towns (OR = 1.85, 95% CI: 1.05-3.27). Boys, relative to girls, were less likely to meet the recommendation of 6 or more servings of vegetables and fruit per day (Table 2).

## Discussion

In the present study we revealed gender-differences in physical activity and nutrition behaviors, the established causes of overweight. Girls reported to be less engaged in physical activities whereas boys reported unhealthier diets. Gender frameworks are not only important to our understanding of the determinants of health, but also for the development of effective health promotion programs [33]. Our results support the development of gender-focused health promotion programs as a strategy to reduce the burden of overweight and consequent chronic diseases.

### Gender and overweight

We reported a slightly higher overweight prevalence among boys than girls. This observation is consistent with other Canadian and US studies that reported slightly lower prevalence of overweight among girls [11,34-37]. When focusing on specific geographies, we found that boys residing in towns were substantially more likely to be overweight as compared to girls. This reinforces the need to considering geographic discrepancies in addition to gender-focused approaches in the development of preventive strategies.

### Gender and health behaviors

Various studies have shown that boys engaged more in physical activity than girls [38-40]. Physical activity counterbalances excess energy intake and the risk for overweight. This risk seems to be particularly present in rural schools and calls for programs to promote physical activity, particularly among girls during their transition from childhood to adolescence and adulthood, as longitudinal studies have shown a drop in activity levels during these years [5]. Specifically, a review of the literature suggested annual declines in physical activity of up to 2.7% among boys and 7.4% among girls between the ages of 10-17 years [5].

Our study population constitutes grade five students who are primarily 10 or 11 years old and primarily preadolescent. Adolescence is a period of physical, psychosocial, cognitive, and emotional change that may be linked to physical maturity and may affect physical activity behaviors [41]. Thomson et al. [42] demonstrated that gender differences in physical activity diminished when aligned on maturational age, suggesting that sexual maturation may be intricately involved in the adolescent decline in physical activity. As girls mature approximately 2 years before boys [43], relative more girls than boys are expected to have entered sexual maturation in our study population. This may have contributed to the higher physical activity levels among boys relative to girls observed in this study. A US-based study suggested that early-maturing girls are twice as likely to be obese compared with early-maturing boys [44]. If this were to apply to our study participants, we should anticipate changing gender differentials in overweight in the year to come.

We observed gender-difference in dietary intake and eating behaviors that parallel observations in the relatively few studies that focused on gender and nutrition among children and adolescents [4,34,36,45,46]. We observed higher calorie intakes among boys relative to girls, which is consistent with another Canadian study [4] and the fact that energy requirements among boys are slightly higher than among girls [37], though are inconsistent with studies reporting an absence in gender difference in children's energy intake [45,47]. The limited number of regarding gender comparisons in the literature likely reflects the challenge of collecting and analyzing dietary information among children.

### The need for gender-focused interventions

Differences in overweight and health behaviors between boys and girls may result from differences in biology (sex differences), from differences assumed to be due to society or culture (gender differences), or a combination of the two [6,48]. With respect to health in general, there is support that integrating gender considerations into interventions will improve their effectiveness [24], though it is acknowledged that these gender considerations are often difficult to translate into health promotion and preventive programs [49]. In the Canadian public health care system, patient care absorbs the far majority of health sector resources, with less than 3% of health spending allocated for resources towards health promotion and primary prevention [50]. Therefore it is of utmost importance to invest these limited resources in effective preventive activities [51]. The lack of translation of knowledge about gender differentials in health and health behaviors into health promotion interventions may lead to a weakened potential for success and herewith inefficient use of resources [52]. Thus, studies into gender differences are important to increase our understanding of mechanisms of behavioral change to allow optimizing health promotion and interventions [53]. The present study contributes to this understanding for the prevention of childhood overweight and obesity. The results reinforce the needs for gender-focused health promotion and provide direction on how to deliver that.

Strengths of the present study include its large representative sample, measured heights and weights and its response rate that is considered high for school-based research. A further limitation relates to the cross-sectional design that necessitates caution with respect to interpretations related to directionality and causality. We had used the body mass index cut-off values by the International Obesity Task Force to define overweight and obesity [27]. These are increasingly established and some studies showed that they provided similar definitions for overweight though more conservative estimates for obesity [54-57].

Self report is prone to error. As in other studies, in the present study this may have lead to an over-reporting of "structured" activities and sport activities [41]. The use of objective measures of physical activity (e.g. pedometers) would have permitted us to more accurately estimate physical activity levels of students, although it is acknowledged that the use of such devices may be logistically and financially challenging in large population studies. The limitations of self report also apply to the assessment of dietary intake, although the Harvard Food Frequency Questionnaire for Youth and Adolescents is a extensively validated and used instrument [58]. Studies have shown that FFQ tend to underestimate energy intake when compared with measured energy intake [59,60].

## Conclusions

To conclude, this study highlights gender-differences in physical activity and nutrition behaviors, the underlying causes of overweight, in a large representative sample of Canadian preadolescent children. The results add to our understanding of the importance of gender in primary prevention of chronic disease. While overall gender-differences in overweight prevalence are relative small, gender-differences in health behavior are apparent and will affect the development of eating and activity habits if not intervened upon. Recognizing gender inequalities may allow for the development of more effective health promotion strategies. Without such gender considerations in health promotion, gender inequalities may in fact enhance. Where internationally considerable work is underway to integrate gender perspectives into preventative policies, the present study identified for a Canadian setting that physical activity among girls and healthy eating among boys are priority areas.

## Competing interests

The authors declare that they have no competing interests.

## Authors' contributions

ASK conducted the statistical analysis and literature review, and drafted the manuscript. PJV is the principal investigator, advised on the analytic approaches and assisted in the manuscript writing. Both authors read and approved the final version.

## Pre-publication history

The pre-publication history for this paper can be accessed here:

http://www.biomedcentral.com/1471-2458/10/340/prepub

## References

[B1] Lobstein T Baur LA Uauy R Obesity in children and young people: a crisis in public health Obes Rev 2004 5 Suppl 1 4 104 10.1111/j.1467-789X.2004.00133.x 15096099

[B2] Shields M Overweight and obesity among children and youth Health Rep 2006 17 27 42 16981484

[B3] Raine KD Determinants of healthy eating in Canada: an overview and synthesis Can J Public Health 2005 96 Suppl 3 S8 15 16042158 10.1007/BF03405195 PMC6976210

[B4] Veugelers PJ Fitzgerald AL Prevalence of and risk factors for childhood overweight and obesity Can Med Ass J 2005 173 607 613 10.1503/cmaj.050445 PMC1197160 16157724

[B5] Sweeting HN Gendered dimensions of obesity in childhood and adolescence Nutr J 2008 14 7:1 10.1186/1475-2891-7-1 PMC2265740 18194542

[B6] Krieger N Genders, sexes, and health: what are the connections - and why does it matter? Int J Epidemiol 2003 32 652 657 10.1093/ije/dyg156 12913047

[B7] Skelton JA Cook SR Auinger P Klein JD Barlow SE Prevalence and trends of severe obesity among US children and adolescents Acad Pediatr 2009 9 322 329 10.1016/j.acap.2009.04.005 19560993 PMC2746875

[B8] Munakata H Sei M Ewis AA Umeno M Sato Y Nakano T Sakamoto K Yoshida Y Onishi C Nakahori Y rediction of Japanese children at risk for complications of childhood obesity: gender differences for intervention approaches J Med Invest 2010 57 62 68 10.2152/jmi.57.62 20299744

[B9] Wisniewski AB Chernausek SD Gender in childhood obesity: family environment, hormones, and genes Gend Med 2009 6 Suppl 1 76 85 Review 10.1016/j.genm.2008.12.001 19318220

[B10] Tremblay MS Katzmarzyk PT Willms JD Temporal trends in overweight and obesity in Canada, 1981-1996 Int J Obes Relat Metab Disord 2002 26 538 543 10.1038/sj.ijo.0801923 12075581

[B11] Baskin ML Ard J Franklin F Allison DB Prevalence of obesity in the United States Obes Rev 2005 6 5 7 10.1111/j.1467-789X.2005.00165.x 15655032

[B12] Watkins DC Murray LJ McCarron P Boreham CA Cran GW Young IS McGartland C Robson PJ Savage JM Ten-year trends for fatness in Northern Irish adolescents: the Young Hearts Projects--repeat cross-sectional study Int J Obes (Lond) 2005 29 579 585 10.1038/sj.ijo.0802945 15889116

[B13] Xie B Chou CP Spruijt-Metz D Reynolds K Clark F Palmer PH Gallaher P Sun P Guo Q Johnson CA Socio-demographic and economic correlates of overweight status in Chinese adolescents Am J Health Behav 2007 31 339 352 17511569 10.5555/ajhb.2007.31.4.339

[B14] Salazar-Martinez E Allen B Fernandez-Ortega C Torres-Mejia G Galal O Lazcano-Ponce E Overweight and obesity status among adolescents from Mexico and Egypt Arch Med Res 2006 37 535 542 10.1016/j.arcmed.2005.10.014 16624655

[B15] Bénéfice E Garnier D Simondon KB Malina RM Relationship between stunting in infancy and growth and fat distribution during adolescence in Senegalese girls Eur J Clin Nutr 2001 55 50 58 10.1038/sj.ejcn.1601121 11303494

[B16] Lorson BA Melgar-Quinonez HR Taylor CA Correlates of fruit and vegetable intakes in US children J Am Diet Ass 2009 109 474 478 10.1016/j.jada.2008.11.022 19248865

[B17] Galloway T Gender differences in growth and nutrition in a sample of rural Ontario schoolchildren Am J Hum Biol 2007 19 774 788 10.1002/ajhb.20637 17676611

[B18] Caine-Bish NL Scheule B Gender differences in food preferences of school-aged children and adolescents J Sch Health 2009 79 532 540 10.1111/j.1746-1561.2009.00445.x 19840230

[B19] Al Sabbah H Vereecken C Kolsteren P Abdeen Z Maes L Food habits and physical activity patterns among Palestinian adolescents: findings from the national study of Palestinian schoolchildren (HBSC-WBG2004) Public Health Nutr 2007 10 739 46 10.1017/S1368980007665501 17381946

[B20] Irving HM Adlaf EM Allison KR Paglia A Dwyer JJ Goodman J Trends in vigorous physical activity participation among Ontario adolescents, 1997-2001 Can J Public Health 2003 94 272 274 12873085 10.1007/BF03403550 PMC6980256

[B21] Kirchengast S Marosi A Gender differences in body composition, physical activity, eating behavior and body image among normal weight adolescents--an evolutionary approach Coll Antropol 2008 32 1079 86 19149211

[B22] Hoelscher DM Barroso C Springer A Castrucci B Kelder SH Prevalence of self-reported activity and sedentary behaviors among 4th-, 8th-, and 11th-grade Texas public school children: the school physical activity and nutrition study J Phys Act Health 2009 6 535 547 19953830 10.1123/jpah.6.5.535

[B23] De Cocker K Ottevaere C Sjöström M Moreno LA Wärnberg J Valtueña J Manios Y Dietrich S Mauro B Artero EG Molnár D Hagströmer M Ruiz JR Sarri K Kafatos A Gottrand F De Henauw S Maes L De Bourdeaudhuij I Self-reported physical activity in European adolescents: results from the HELENA (Healthy Lifestyle in Europe by Nutrition in Adolescence) study Public Health Nutr 2010 in press 20236565 10.1017/S1368980010000558

[B24] Brown T Summerbell C Systematic review of school-based interventions that focus on changing dietary intake and physical activity levels to prevent childhood obesity: an update to the obesity guidance produced by the National Institute for Health and Clinical Excellence Obes Rev 2009 10 110 141 10.1111/j.1467-789X.2008.00515.x 18673306

[B25] Alberta Education Student population by grade, school and authority, Alberta 2006/2007 school year http://education.alberta.ca/apps/eireports/pdf_files/eis1004_2007/eis1004_2007.pdf Last accessed November 15, 2007

[B26] Rockett HR Wolf AM Colditz GA Development and reproducibility of a food frequency questionnaire to assess diets of older children and adolescents J Am Diet Assoc 1995 95 336 40 10.1016/S0002-8223(95)00086-0 7860946

[B27] Cole TJ Bellizzi MC Flegal KM Dietz WH Establishing a standard definition for child overweight and obesity worldwide: international survey BMJ 2000 320 1240 1243 10.1136/bmj.320.7244.1240 10797032 PMC27365

[B28] Crocker PRE Bailey DA Faulkner RA Kowalski KC McGrath R Measuring general levels of physical activity: Preliminary evidence for the Physical Activity Questionnaire for Older Children Med Sci Sports Exerc 1997 29 1344 1349 9346166 10.1097/00005768-199710000-00011

[B29] Statistics Canada The National Longitudinal Survey among Children and Youth (NSLCY) http://www.statcan.gc.ca/imdb-bmdi/4450-eng.htm Last accessed October 20, 2009

[B30] Health Canada Canadian Nutrient File (CNF) 2007 http://www.hc-sc.gc.ca/fn-an/nutrition/fiche-nutri-data/index-eng.php Last accessed September 2009

[B31] Government of Canada Canadian Food Guide http://www.hc-sc.gc.ca/fn-an/alt_formats/hpfb-dgpsa/pdf/food-guide-aliment/print_eatwell_bienmang-eng.pdf Last accessed September 2009

[B32] Willett W Nutritional Epidemiology 1998 2 New York: Oxford University Press

[B33] Keleher H Why build a health promotion evidence base about gender? Health Promot Int 2004 19 277 279 10.1093/heapro/dah313 15306611

[B34] Tremblay MS Willms JD Secular trends in the body mass index of Canadian children Can Med Ass J 2000 163 1429 1433 PMC80409 11192647

[B35] Hanning RM Woodruff SJ Lambraki I Jessup L Driezen P Murphy CC Nutrient intakes and food consumption patterns among Ontario students in grades six, seven, and eight Can J Public Health 2007 98 12 16 17278670 10.1007/BF03405377 PMC6976020

[B36] Hedley AA Ogden CL Johnson CL Carroll MD Curtin LR Flegal KM Prevalence of overweight and obesity among US children, adolescents, and adults, 1999-2002 JAMA 2004 291 2847 2850 10.1001/jama.291.23.2847 15199035

[B37] Galloway T Gender differences in growth and nutrition in a sample of rural Ontario schoolchildren Am J Hum Biol 2007 19 774 788 10.1002/ajhb.20637 17676611

[B38] Botton J Heude B Kettaneh A Borys JM Lommez A Bresson JL Ducimetiere P Charles MA FLVS Study Group Cardiovascular risk factor levels and their relationships with overweight and fat distribution in children: the Fleurbaix Laventie Ville Santé II study Metabolism 2007 56 614 622 10.1016/j.metabol.2006.12.006 17445535 PMC1988890

[B39] Sherar LB Esliger DW Baxter-Jones AD Tremblay MS Age and gender differences in youth physical activity: does physical maturity matter? Med Sci Sports Exerc 2007 39 830 835 10.1249/mss.0b013e3180335c3c 17468582

[B40] Storey KE Forbes LE Fraser SN Spence JC Plotnikoff RC Raine KD Hanning RM McCargar LJ Diet quality, nutrition and physical activity among adolescents: the Web-SPAN (Web-Survey of Physical Activity and Nutrition) project Public Health Nutr 2009 12 2009 2017 10.1017/S1368980009990292 19545471

[B41] Sallis JF Saelens BE Assessment of physical activity by self-report: status, limitations, and future directions Res Q Exerc Sport 2000 71 2 Suppl S1 14 Review 10925819

[B42] Thomson AM Baxter-Jones AD Mirwald RL Bailey DA Comparison of physical activity in male and female children: does maturation matter? Med Sci Sports Exerc 2005 37 1902 1908 10.1249/01.mss.0000176306.11134.23 14523305

[B43] Malina RM Eisenmann JC Cumming SP Ribeiro B Aroso J Maturity-associated variation in the growth and functional capacities of youth football (soccer) players 13-15 years Eur J Appl Physiol 2004 91 555 562 10.1007/s00421-003-0995-z 14648128

[B44] Wang Y Is obesity associated with early sexual maturation? A comparison of the association in American boys versus girls Pediatrics 2002 110 903 910 10.1542/peds.110.5.903 12415028

[B45] Champagne CM Bogle ML Karge WH Using national dietary data to measure dietary changes Public Health Nutr 2002 5 985 10.1079/PHN2002375 12633523

[B46] Moffat T Galloway T Latham J Stature and adiposity among children in contrasting neighborhoods in the city of Hamilton, Ontario, Canada Am J Hum Biol 2005 17 355 367 10.1002/ajhb.20125 15849705

[B47] Cooke L Grant M Support for evidence-based practice Semin Oncol Nurs 2002 18 71 78 10.1053/sonu.2002.30049 11878043

[B48] Bird CE Rieker PP Gender matters: an integrated model for understanding men's and women's health Soc Sci Med 1999 48 745 755 10.1016/S0277-9536(98)00402-X 10190637

[B49] Ostlin P Mackenbach J and Bakker M Gender perspective on socio-economic inequalities in Health Reducing inequalities in Health 2002 London, Routledge 25 49

[B50] Hylton J Improving Health Status through Intersectorial cooperation 2003 Muttart Foundation, Edmonton, Canada

[B51] Ostlin P Eckermann E Mishra US Nkowane M Wallstam E Gender and health promotion: a multisectoral policy approach Health Promot Int 2006 21 25 35 10.1093/heapro/dal048 17307954

[B52] Thomas H Obesity prevention programs for children and youth: why are their results so modest? Health Educ Res 2006 21 783 795 10.1093/her/cyl143 17099075

[B53] Kremers SP de Bruijn GJ Droomers M van Lenthe F Brug J Moderators of environmental intervention effects on diet and activity in youth Am J Prev Med 2007 32 163 172 10.1016/j.amepre.2006.10.006 17197152

[B54] Shields M Tremblay MS Canadian childhood obesity estimates based on WHO, IOTF and CDC cut-points Int J Pediatr Obes 2010 5 3 265 73 10.3109/17477160903268282 20210678

[B55] Edwards J Evans J Brown AD Using routine growth data to determine overweight and obesity prevalence estimates in preschool children in the Capital Health Region of Alberta Can J Public Health 2008 99 91 94 18457279 10.1007/BF03405451 PMC6975785

[B56] Wang Y Wang JQ A comparison of international references for the assessment of child and adolescent overweight and obesity in different populations Eur J Clin Nutr 2002 56 973 982 10.1038/sj.ejcn.1601415 12373618

[B57] Kain J Uauy R Vio F Albala C Trends in overweight and obesity prevalence in Chilean children: comparison of three definitions Eur J Clin Nutr 2002 56 200 204 10.1038/sj.ejcn.1601301 11960294

[B58] Rockett HR Breitenbach M Frazier AL Witschi J Wolf AM Field AE Colditz GA Validation of a youth/adolescent food frequency questionnaire Prev Med 1997 26 808 816 10.1006/pmed.1997.0200 9388792

[B59] Schaefer EJ Augustin JL Schaefer MM Rasmussen H Ordovas JM Dallal GE Dwyer JT Lack of efficacy of a food-frequency questionnaire in assessing dietary macronutrient intakes in subjects consuming diets of known composition Am J Clin Nutr 2000 71 746 51 10702168 10.1093/ajcn/71.3.746

[B60] Paul DR Rhodes DG Kramer M Baer DJ Rumpler WV Validation of a food frequency questionnaire by direct measurement of habitual ad libitum food intake Am J Epidemiol 2005 162 806 814 10.1093/aje/kwi279 16120695

